# Ranula vs. Atypical Sublingual Branchial Cleft Cyst: A Case Report

**DOI:** 10.1002/ccr3.72208

**Published:** 2026-04-09

**Authors:** Saleh Mohebbi, Fatemeh Abedin, Sahar Zahedi, Mehdi Abrishami

**Affiliations:** ^1^ Skull Base Research Center, School of Medicine Iran University of Medical Sciences Tehran Iran; ^2^ ENT and Head and Neck Research Center The Five Sense Health Institute, Iran University of Medical Sciences Tehran Iran; ^3^ Department of Oral & Maxillofacial Surgery, Faculty of Dentistry Isf.C, Islamic Azad University, University Boulevard Isfahan Iran

**Keywords:** branchial cleft cyst, histopathology, neck mass, ranula, surgery, thyroglossal duct cyst

## Abstract

Congenital cystic lesions in the floor of the mouth in infants present significant diagnostic challenges owing to overlapping clinical and radiological features. We report a rare case of a one‐year‐old boy with a gradually enlarging, painless left sublingual swelling noticed since birth. Clinical examination revealed a soft, fluctuant 3 × 4 cm mass that did not move with deglutition or tongue protrusion, along with a small midline submental dimple. Contrast‐enhanced computed tomography (CECT) showed a well‐defined, non‐enhancing cystic lesion (4.1 × 2.1 × 3.9 cm) in the left sublingual space, compressing adjacent muscles without extension to the hyoid bone or submandibular gland. A provisional diagnosis of plunging ranula was made preoperatively. The lesion was completely excised via an intraoral approach. Intraoperatively, thick yellow fluid and a fistulous tract extending toward the submental dimple were noted. Histopathology demonstrated a cyst lined by pseudostratified columnar respiratory epithelium in some areas and stratified squamous epithelium in others, with no thyroid tissue present—findings consistent with an atypical sublingual branchial cleft cyst (likely of second branchial pouch origin) associated with a sinus tract rather than a simple or plunging ranula. The postoperative course was uneventful, with no recurrence at six‐month follow‐up. This case underscores the rarity of branchial cleft remnants manifesting as isolated sublingual cysts in infancy and highlights the importance of complete surgical excision, including any associated sinus tract, to prevent recurrence. It also illustrates the limitations of preoperative imaging in definitively distinguishing ranula from atypical branchial anomalies in young children.

## Key Clinical Message

Congenital sublingual cystic swellings in infants may rarely represent atypical branchial cleft cysts rather than a ranula. The presence of a midline submental dimple/sinus tract is a crucial clinical clue. Complete excision, including the tract, is essential to prevent recurrence, as preoperative imaging may be misleading.

## 1 Introduction

Lesions occurring in the floor of the mouth often present significant diagnostic challenges due to the complex anatomical structure of the region and the overlapping clinical manifestations of various pathologies. Among the most common cystic lesions in this area is a ranula. However, branchial cleft cysts, particularly when they arise in atypical locations, should be considered. Misdiagnosis of these lesions can lead to inappropriate treatment strategies, remnant, and recurrence. Therefore, a comprehensive understanding of their pathophysiology, clinical features, imaging characteristics, and histopathology is essential for accurate differential diagnosis and appropriate surgical decision‐making [1, 2].

Ranulas are mucous pseudocysts typically resulting from trauma or obstruction of the sublingual gland ducts, causing mucin extravasation into surrounding tissues. They are classified as simple (oral) ranulas, confined to the sublingual space, or plunging ranulas, which extend through the mylohyoid muscle into the neck. Histologically, ranulas lack a true epithelial lining and are surrounded by granulation tissue, distinguishing them from true cysts [3].

The sublingual glands, with their multiple ductal openings and continuous basal secretion, are more susceptible to ranula formation than the submandibular glands. While typical ranulas are often diagnosed clinically, imaging—particularly magnetic resonance imaging (MRI)—is vital for assessing extent and identifying plunging variants. Branchial cleft cysts, in contrast, are congenital anomalies arising from incomplete obliteration of the branchial apparatus during embryonic development. Second branchial cleft cysts, the most common type, typically present in the lateral neck; however, rare cases occur in the floor of the mouth and are frequently misdiagnosed as dermoid cysts, lymphangiomas, or ranulas [2, 3].

Sublingual branchial cleft cysts are extremely rare, with only a handful of cases documented in the literature since the first report by Robins in 1969 [4]. These atypical presentations frequently mimic ranulas clinically and radiologically, highlighting the diagnostic challenge and the value of including such entities in the differential diagnosis [5, 6].

Differentiation between ranulas and sublingual branchial cleft cysts is particularly challenging when the lesion appears as a soft, nonspecific swelling without cervical extension or distinctive imaging features. The differential should also encompass dermoid/epidermoid cysts, lymphangiomas, and cystic salivary gland tumors. MRI provides superior soft‐tissue contrast; ranulas characteristically appear homogeneous with high T2 signal intensity and may show the “tail sign”—a tapering extension toward the sublingual gland [3, 4].

Preoperative fine‐needle aspiration (FNA) is sometimes performed but often yields nonspecific results due to the mucinous content in ranulas or benign epithelial lining in branchial cysts. Ultimately, complete surgical excision followed by histopathological examination remains the gold standard for definitive diagnosis.

Treatment approaches differ markedly: for ranulas, excision of the sublingual gland is the gold standard to eliminate the mucin source and minimize recurrence, while marsupialization or simple excision carries a higher recurrence risk. Plunging ranulas may require a transcervical approach [1, 4].

Branchial cleft cysts, however, necessitate complete excision of the cyst wall and any associated fistulous tract to prevent recurrence or infection; incomplete removal can lead to persistent discharge or regrowth [4].

Case reports elucidating the diagnostic ambiguity between ranulas and atypically located branchial cleft cysts are valuable additions to the medical literature. By integrating clinical, radiological, and histopathological findings, such reports help refine diagnostic algorithms and surgical strategies for these rare entities.

## 2 Case History and Examination

A one‐year‐old boy was brought by his parents with a history of gradually progressive, painless swelling in the left floor of the mouth, first noticed by his parents since birth (early infancy). The swelling had been present from birth but remained stable initially and was managed conservatively with observation. Over the subsequent months, it enlarged slowly without any associated symptoms. There was no history of trauma, infection, dysphagia, respiratory distress, feeding difficulties, or recurrent swelling. Prenatal, perinatal, and past medical history were unremarkable, with no significant family history of congenital anomalies.

**FIGURE 1:**
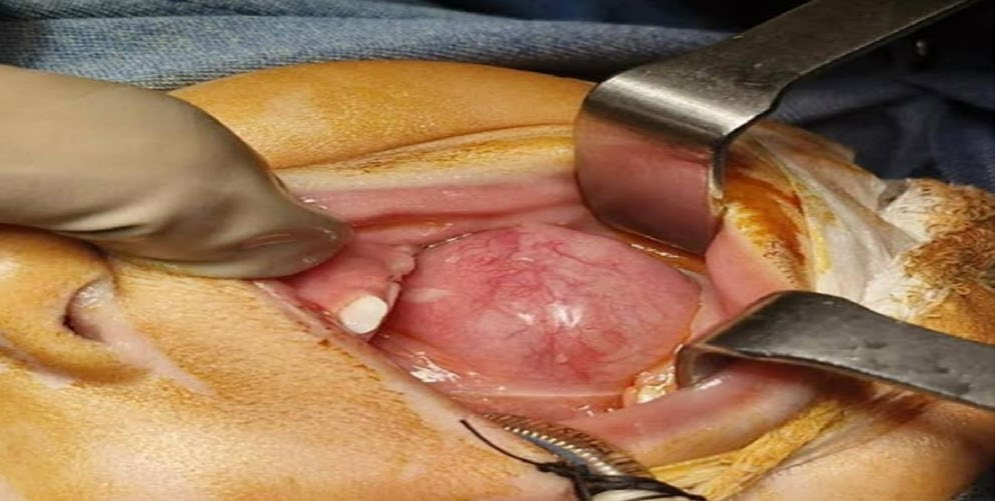
Preoperative view showing diffuse, non‐tender swelling in the left sublingual region.

On clinical examination (at presentation at 1 year of age), a solitary, diffuse, soft, fluctuant, non‐tender swelling measuring approximately 3 × 4 cm was noted in the left sublingual region (Figure 1). The overlying mucosa appeared normal. Importantly, the mass did not move with deglutition or tongue protrusion, ruling out a thyroglossal duct remnant. Additionally, a small midline dimple/opening was observed on the chin in the submental region, without discharge or inflammation, or associated with a sinus tract externally. No cervical lymphadenopathy or other head and neck abnormalities were detected.

## 3 Differential Diagnosis

The main differential diagnoses considered were as follows:

- Plunging ranula (mucocele of the sublingual gland)
- Sublingual dermoid/epidermoid cyst
- Lymphatic malformation (cystic hygroma)
- Thyroglossal duct cyst (atypical location)
- Branchial cleft cyst (second or atypical branchial anomaly presenting in the sublingual/submental region)
- Foregut duplication cyst
- Sublingual abscess (less likely due to absence of pain, fever, or inflammatory signs)

## 4 Investigations

**FIGURE 2:**
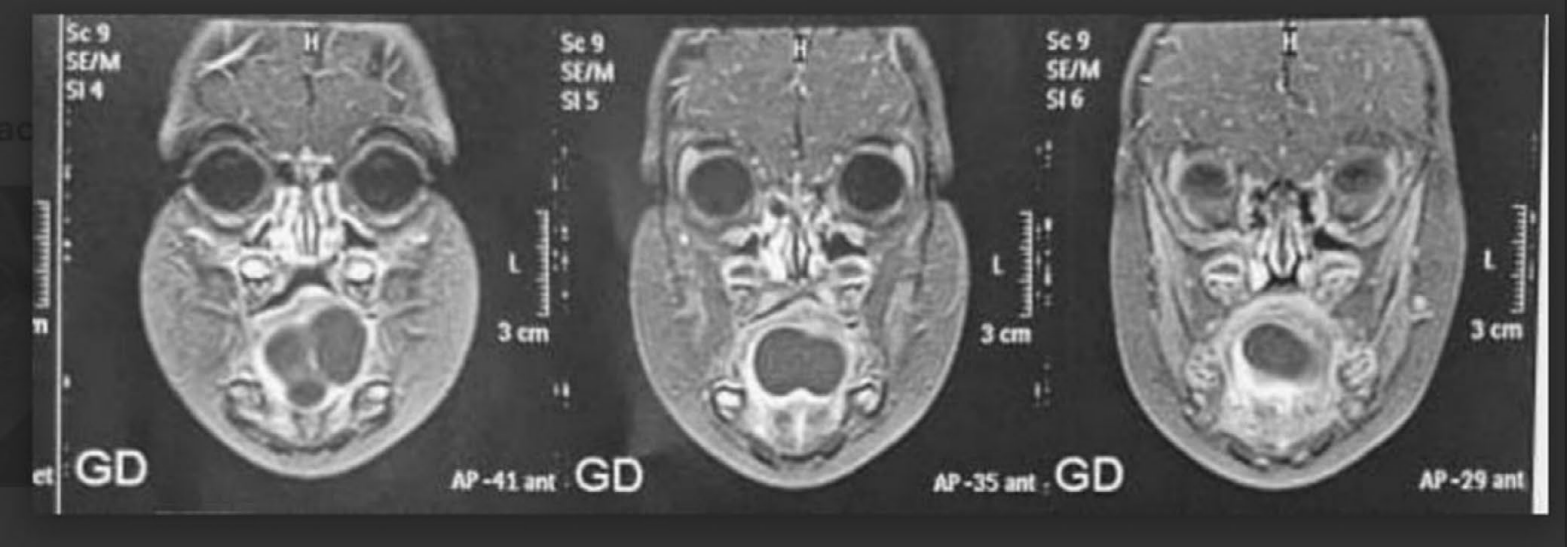
CECT image showing a well‐defined, hypodense cystic lesion in the sublingual space.

Contrast‐enhanced computed tomography (CECT) of the neck (performed preoperatively) revealed a well‐defined, unilocular, hypodense, non‐enhancing cystic lesion in the left sublingual space measuring 4.1 × 2.1 × 3.9 cm (Figure 2). The lesion caused compression and displacement of the left genioglossus and hyoglossus muscles but showed no extension to the base of the tongue or hyoid bone. There was no solid component, calcification, or significant enhancement, and no evident connection with the submandibular gland.

Fine‐needle aspiration cytology (FNAC) was planned but could not be performed due to poor cooperation of the child and the risk of cyst rupture or seeding.

Based on clinical and radiological findings, a provisional preoperative diagnosis of plunging ranula was made.

## 5 Treatment, Outcome, and Follow‐Up

Surgical excision was performed under general anesthesia via an intraoral approach. Intraoperatively, the lesion was submucosal, cystic, and contained thick, yellow, pus‐like fluid (later confirmed as mucoid material with inflammatory cells). A thin fistulous tract was identified extending from the cyst toward the midline submental region, corresponding to the external dimple noted clinically. Complete excision of the cyst along with the entire fistulous tract was achieved. The sublingual gland was not removed.

Histopathological examination showed a cyst lined by pseudostratified columnar respiratory epithelium in some areas and stratified squamous epithelium in others, with focal areas of chronic inflammation. No thyroid tissue or cartilage was identified. These findings were more consistent with an atypical second branchial cleft cyst (or branchial pouch remnant) with a sinus tract rather than a simple ranula or mucocele.

The postoperative course was uneventful. The child resumed oral feeds on the first postoperative day and was discharged on the second postoperative day. Regular follow‐up over the subsequent 6 months showed complete healing, with no evidence of recurrence, scarring, or functional impairment.

## 6 Discussion

The management of this case highlighted several strengths, including the use of intraoperative imaging to accurately delineate the anatomical extent of the sublingual cystic lesion, thereby facilitating precise surgical planning through an intraoral approach. Furthermore, the intraoperative identification and tracing of a fistulous tract enabled thorough excision, minimizing the risk of recurrence.

Sublingual branchial cleft cysts are exceptionally rare, with only a limited number of cases documented in the literature. In contrast to typical second‐branchial cleft cysts, which commonly present as painless swellings in the lateral neck, anterior to the sternocleidomastoid muscle, and near the angle of the mandible [7, 8], the sublingual location in our case represents a highly atypical presentation, contributing to the diagnostic challenge. As noted by Sutton and Goldman [9], branchial cleft cysts exhibit an epithelial lining composed of stratified squamous epithelium (with occasional ciliated columnar variants) surrounded by lymphoid tissue, which is consistent with our findings. Panchbhai et al. [10] reported a sublingual branchial cleft cyst mimicking a ranula, underscoring the diagnostic challenge due to overlapping clinical presentations with ranulas, thyroglossal duct cysts (TGDCs), and dermoid cysts [1].

Unlike TGDCs, which typically move with tongue protrusion, sublingual branchial cysts lack this mobility, as observed in our case and supported by Voss et al. [11]. Preoperative imaging, particularly CECT or MRI, is critical for anatomical delineation and differential diagnosis, as highlighted by Baba et al. [12] and Choi et al. [13]. Surgical excision remains the definitive treatment, with complete resection essential to prevent recurrence, as demonstrated in our case and corroborated by studies reporting low recurrence rates with en bloc resection [14]. The identification of a fistulous tract in our case aligns with reports by Chen et al. [6] who described fistulous connections in first branchial cleft anomalies, emphasizing the need for meticulous intraoperative exploration.

This case emphasizes the importance of including sublingual branchial cleft cysts in the differential diagnosis of floor‐of‐mouth masses, despite their rarity. Key lessons learned include the critical role of preoperative imaging for anatomical delineation, the necessity of intraoperative vigilance to identify associated fistulous tracts, and the reliance on histopathological examination for definitive diagnosis. Clinicians should maintain a high index of suspicion for atypical presentations, as misdiagnosis with ranulas, branchial cleft cysts, or TGDCs can lead to inappropriate management. Complete surgical excision via an intraoral approach is effective and curative, provided adjacent neurovascular structures are preserved. Multidisciplinary collaboration involving radiologists, pathologists, and surgeons is essential to optimize outcomes in such rare cases.

Histopathological confirmation was pivotal in refining the diagnosis and distinguishing the lesion from other differentials. However, limitations included the inability to perform fine‐needle aspiration cytology (FNAC) due to the cystic nature of the mass and the young age of the patient. Additionally, the absence of advanced imaging modalities such as MRI may have limited a more detailed assessment of soft‐tissue relationships.

## 7 Conclusions

In conclusion, although sublingual branchial cleft cysts are rare, they should be considered in the differential diagnosis of midline floor‐of‐mouth masses in infants. Accurate diagnosis relies on histopathological confirmation, supported by imaging to guide surgical planning. Complete excision is curative, with low recurrence rates when the cyst and any associated tract are fully removed. This case highlights the need for heightened clinical suspicion and multidisciplinary collaboration in managing these atypical congenital anomalies.

## Author Contributions

**Saleh Mohebbi:** conceptualization, project administration. **Fatemeh Abedin:** investigation, methodology. **Sahar Zahedi:** project administration, validation. **Mehdi Abrishami:** writing – original draft, writing – review and editing.

## Funding

The authors have nothing to report.

## Consent

Written informed consent was obtained from the patient's parents for the publication of this case report, including clinical details, images, and accompanying photographs. A copy of the signed consent form is available for review by the Editor‐in‐Chief upon request. The parents were fully informed about the purpose of publication and assured that the child's identity would remain anonymous.

## Conflicts of Interest

The authors declare no conflicts of interest.

## Data Availability

The data that support the findings of this study are available from the corresponding author upon reasonable request.
